# Serum Uric Acid and Adiposity: Deciphering Causality Using a Bidirectional Mendelian Randomization Approach

**DOI:** 10.1371/journal.pone.0039321

**Published:** 2012-06-19

**Authors:** Tanica Lyngdoh, Philippe Vuistiner, Pedro Marques-Vidal, Valentin Rousson, Gérard Waeber, Peter Vollenweider, Murielle Bochud

**Affiliations:** 1 Institute of Social and Preventive Medicine (IUMSP), Lausanne University Hospital, Lausanne, Switzerland; 2 Department of Medicine, Internal Medicine, CHUV, Lausanne, Switzerland; Innsbruck Medical University, Austria

## Abstract

**Background:**

Although the relationship between serum uric acid (SUA) and adiposity is well established, the direction of the causality is still unclear in the presence of conflicting evidences. We used a bidirectional Mendelian randomization approach to explore the nature and direction of causality between SUA and adiposity in a population-based study of Caucasians aged 35 to 75 years.

**Methods and Findings:**

We used, as instrumental variables, *rs6855911* within the SUA gene *SLC2A9* in one direction, and combinations of SNPs within the adiposity genes *FTO*, *MC4R* and *TMEM18* in the other direction. Adiposity markers included weight, body mass index, waist circumference and fat mass. We applied a two-stage least squares regression: a regression of SUA/adiposity markers on our instruments in the first stage and a regression of the response of interest on the fitted values from the first stage regression in the second stage. SUA explained by the *SLC2A9* instrument was not associated to fat mass (regression coefficient [95% confidence interval]: 0.05 [−0.10, 0.19] for fat mass) contrasting with the ordinary least square estimate (0.37 [0.34, 0.40]). By contrast, fat mass explained by genetic variants of the *FTO*, *MC4R* and *TMEM18* genes was positively and significantly associated to SUA (0.31 [0.01, 0.62]), similar to the ordinary least square estimate (0.27 [0.25, 0.29]). Results were similar for the other adiposity markers.

**Conclusions:**

Using a bidirectional Mendelian randomization approach in adult Caucasians, our findings suggest that elevated SUA is a consequence rather than a cause of adiposity.

## Introduction

High serum uric acid (SUA) is known to co-exist with the components of metabolic syndrome including obesity [1]–[3]. Epidemiological studies found positive associations between SUA and different adiposity markers including waist circumference [4], body mass index (BMI) [4], waist-to-hip ratio [5] and body fat [6], [7]. Although the relationship between SUA and adiposity appears to be well-established in conventional observational analysis, it is difficult to ascertain if these associations are truly causal or are a consequence of bias or residual confounding. Further, the relationship between SUA and adiposity is complicated by evidence suggesting the possibility of causality in both directions.

Some hypothesized that SUA mediates obesity and other features of metabolic syndrome by reducing endothelial nitric oxide and decreasing insulin-mediated glucose uptake in skeletal muscle [8]. Several pieces of evidence are in line with this direction of causality. In longitudinal epidemiologic studies, baseline SUA independently predicted weight gain [9], the development of impaired fasting glucose [10] or incident type 2 diabetes [10]–[13], even in the absence of metabolic syndrome [13] or obesity [9], [10] at baseline. Analogously, baseline hyperuricemia independently predicted 9-year incident hyperinsulinemia in the ARIC cohort [14], which suggests that hyperuricemia is not merely the consequence of hyperinsulinemia. Baseline hyperuricemia was also an independent predictor of 5-year incident metabolic syndrome in a population-based sample in Portugal [15]. Experimental studies have shown that allopurinol, a xanthine oxidase inhibitor that inhibits SUA synthesis, was able to prevent weight gain in fructose-fed rats [16]. Similarly, rats administered uricase inhibitors to induce hyperuricemia, developed features of the metabolic syndrome [17].

Conversely, others suggest that hyperinsulinemia (along with accompanying obesity) reduces urinary uric acid clearance with subsequent elevation of SUA levels [18], [19]. Also, the fact that a genetic risk score robustly associated with SUA was not associated with fasting glucose or insulin levels in the CHARGE consortium speaks against a causal role of uric acid on hyperinsulinemia [20]. Longitudinal epidemiologic studies found baseline BMI [21] or weight gain [22] to predict the development of hyperuricemia during follow-up. Furthermore, weight loss is known to lower SUA levels [23]–[25], which suggests that adiposity leads to hyperuricemia. Hence, further investigations to clarify the nature and direction of the causal link between SUA and adiposity are necessary.

As far as we are aware, the relationship between SUA and adiposity has not been previously explored using the principles of Mendelian randomization, a method that allows disentangling causation from association in the presence of confounding [26]. In a large population-based CoLaus study of Caucasians, we used SUA and adiposity-related genetic variants as instruments in a bidirectional Mendelian randomization approach to explore the links between SUA and adiposity. We performed a Mendelian randomization analysis to determine 1) if adiposity markers such as increased weight, BMI, waist circumference or fat mass are a consequence of elevated SUA or 2) if adiposity leads to hyperuricemia.

SUA is known to have a high (25 to 70%) heritability [27] and recent genome-wide association studies have identified *SLC2A9* to have a strong association with SUA levels [28], [29], explaining about 1.2–6.0% of the variance in SUA concentration [30]. Amongst the adiposity-related genetic variants, we chose single nucleotide polymorphisms (SNPs) within the most common major adiposity genes *FTO, MC4R* and *TMEM18,* all of which have been recognized to be associated with obesity and explaining a variance of about 1–2% [31].

## Materials and Methods

### Study Population

The CoLaus study is a cross-sectional population-based study conducted in Lausanne, Switzerland. Details of the study have been previously described [32]. Briefly, a simple, non-stratified random sample of 19,830 participants, corresponding to 35% of the source population, was drawn, of which 6184 participants were included. Inclusion criteria included a written informed consent, age between 35–75 years and being of Caucasian origin. The study was approved by the Ethics Committee of the University of Lausanne. Recruitment began in June 2003 and ended in May 2006.

### Study Procedure and Measurements

Participants attended the outpatient clinic at Centre Hospitalier Universitaire Vaudois (CHUV) in the morning after an overnight fast. They were asked to continue taking their medication as usual. This examination included detailed questionnaire, physical examination with anthropometric measures by trained and certified field interviewers and laboratory testing. In the present analysis, smoking was defined as present if the participant reported to be current smoker at the time of examination and alcohol consumption was defined as present for participants who report drinking alcohol at least once a day. Diuretic use was assessed by recording all the prescribed drugs taken by the participants and was considered as present if participants were using drugs belonging to any class of diuretics. Height was measured to the nearest 5 mm using a Seca® height gauge (Hamburg, Germany), and weight was measured to the nearest 0.1 kg using a Seca® scale (Hamburg, Germany). These instruments were calibrated regularly. Body mass index was defined as weight divided by height in meter squared. Waist circumference was measured with a non-stretchable tape and the mean of two measurements expressed in centimeters was used for the analyses. Fat mass (in percent of the total body weight) was assessed by electrical bioimpedance using the Bodystat® 1500 analyzer (Isle of Man, British Isles). Fat mass (in kilograms) was calculated from the percentage of fat mass multiplied by weight.

Venous blood samples were collected after an overnight fasting. Most clinical assays were performed by the CHUV Clinical Laboratory on fresh blood samples. Serum creatinine was measured by Jaffe kinetic compensated method (2.9%−1.5% maximum inter and intra-batch coefficients of variation) and uric acid by uricase-PAP (1.0%−0.5%). Glomerular filtration rate (GFR) was estimated using the abbreviated Modification of the Diet in Renal Disease (MDRD) formula: 186×(serum creatinine [µmol/L]/88.4)^(−1.154)^×age^(−0.203)^×F, where F = 1 for men and F = 0.742 for women [33].

### Genotyping

Nuclear DNA was extracted from whole blood for whole genome scan analysis. Genotyping was performed using Affymetrix 500 K SNP chip, as recommended by the manufacturer (Affymetrix, Inc., Santa Clara, California, USA). Persons with less than 95% genotyping efficiency overall (or <90% efficiency on either array; n = 399) and persons with possible gender inconsistencies (n = 5) were removed. Monomorphic SNPs, SNPs with less than 70% genotyping efficiency, SNPs with minor allele frequency less than 1%, and/or not in the Hardy-Weinberg proportions were excluded. A hundred and twenty-nine, 20, 56 and 124 SNPs, 100 kb upstream and downstream of the *FTO*, *MC4R*, *TMEM18* and *SLC2A9* genes respectively, were considered for the present analyses.

### Statistical Analysis

All tests were performed using Stata 11 (StataCorp, College Station, TX, USA). Continuous variables were summarized as mean (standard deviation [SD]) while categorical variables as number of subjects and percentages. We used *t* test and *χ2* test to compare the distribution of covariates according to sex.

Pearson’s partial correlation coefficient test was used to estimate the correlation of SUA with adiposity markers and Fischer’s Z transformation to compare the correlation coefficients between men and women. We performed a bidirectional Mendelian randomization to 1) assess the causality in the direction of SUA causing elevated adiposity and 2) reverse causality i.e. elevated adiposity levels leading to hyperuricemia. In the former, we chose as instrumental variable the SNP with the best F-statistics (Table 1) from the linear regressions between the SNPs within and around the *SLC2A9* gene and SUA level, in the overall sample and separately by sex. We identified *rs6855911*, *rs7442295* and *rs7669607* as the best SNP in the overall sample, men and women respectively. These variants have been identified to be related to SUA in earlier studies [34], [35]. In the latter case, using the SNPs within and around the *FTO*, *MC4R* and *TMEM18* genes separately did not result in strong instruments. To identify sufficiently strong instruments (i.e. an F-statistics >10) [36], we carried out a systematic combination of three SNPs from the three genes separately for each adiposity traits in the overall sample and also by sex. Combinations of four SNPs from the adiposity genes did not lead to significantly better instruments. Based on the genotypes of *FTO*, *MC4R* and *TMEM18*, a score was created for every individual SNP, coded as 0-homozygote for the non-risk allele, 1-heterozygote and 2-homozygote for the risk allele. When combining the SNPs, we summed up their scores using an additive coding for the number of alleles associated with higher adiposity levels. This resulted in an ordinal variable with seven categories coded from 0 to 6. Further, we present the distribution of SUA across genotypes of the *SLC2A9 rs6855911* and adiposity markers across adiposity-related SNPs individually or as genetic scores in the overall sample to see how the specific SNPs relate to the phenotype of interest in the CoLaus participants and used a non-parametric test to assess for trend. In the latter case when using genetic scores to check for trends, we combined participants having scores of 0, 1 and 2 since the number of participants in these categories was small. We also reported the associations of SNP/SNP scores with markers in the hypothesized pathway (i.e. *SLC2A9 rs6855911* with adiposity markers and adiposity-related genetic variants with SUA).

**Table 1:** Association between SNPs chosen as instruments and intermediate phenotype.

		Gene combination			F-statistics	R^2^
		SNP	SNP	SNP		
*Combined SNPs (instruments) within/around FTO, MC4R and TMEM18 for adiposity markers*						
BMI	Overall	*FTO rs1121980*	*FTO rs2665272*	*TMEM18 rs6755502*	27.06	0.0052
	Men	*FTO rs7193144*	*FTO rs6499658*	*TMEM18 rs2683992*	17.59	0.0072
	Women	*FTO rs2540769*	*FTO rs2665272*	*TMEM18 rs2860323*	21.25	0.0083
Fat mass	Overall	*FTO rs7193144*	*FTO rs17823223*	*TMEM18 rs10189761*	28.45	0.0052
	Men	*FTO rs7193144*	*FTO rs16945088*	*FTO rs17823223*	20.73	0.0081
	Women	*FTO rs1121980*	*FTO rs17823223*	*TMEM18 rs7585056*	21.50	0.0076
WC	Overall	*FTO rs1861868*	*FTO rs8050136*	*TMEM18 rs6755502*	36.69	0.0070
	Men	*FTO rs8050136*	*FTO rs8053740*	*MC4R rs17066829*	15.87	0.0061
	Women	*FTO rs1121980*	*FTO rs2665272*	*TMEM18 rs7571872*	31.20	0.0124
Weight	Overall	*FTO rs1121980*	*FTO rs17823223*	*TMEM18 rs6755502*	31.43	0.0060
	Men	*FTO rs7193144*	*FTO rs17823223*	*FTO rs2192872*	16.56	0.0060
	Women	*FTO rs9939973*	*FTO rs836994*	*TMEM18 rs6755502*	21.93	0.0080
*SNPs (instruments) within/around SLC2A9 for SUA*						
SUA	Overall	*SLC2A9 rs6855911*			170.47	0.0316
	Men	*SLC2A9 rs7442295*			71.49	0.0265
	Women	*SLC2A9 rs7669607*			197.21	0.0626

BMI = body mass index; WC = waist circumference; SUA = serum uric acid; SNP = single-nucleotide polymorphism.

To explore the potential causal effect in both directions, we applied a Mendelian randomization approach, also called two-stage least squares (2 SLS) regression, using the instrumental variables that we identified. In the first stage, we conducted an ordinary least square (OLS) regression, regressing SUA/adiposity markers on our instruments (see Table 1 for the choices of instruments in our context). In the second stage, we performed regression of the response of interest (e.g. SUA, BMI, weight etc.) on the fitted values from the first stage regression, which will be referred to as “explained” SUA/adiposity from here on. We conducted the above analysis using the *ivregress* function in Stata 11. To meet the assumptions for linear regression, we used the most appropriate transformations for both the dependent and independent variables (weight and waist: log transformation; SUA and fat mass: square root transformation; and BMI: inverse square root transformation). Further, to facilitate comparability between the coefficients and ease interpretation of the results, both the transformed dependent and independent variables were standardized and results from regression models expressed as 1 SD change in the outcome corresponding to a 1 SD increase in exposure (note that the significance of the results would remain the same without standardization). We tested for interaction by sex using the sex-specific results from the second stage and the following test statistic: (β_men_-β_women_ )/_√_ (S.E_men_^2^+S.E_women_^2^) where β and S.E is the standardized beta coefficient and standard error respectively.

Provided that the assumptions underlying Mendelian randomization are fulfilled, the regression coefficient obtained in the second stage can be interpreted as being the causal effect of the “explained” variable on the response of interest [37]. The first assumption (i.e. the instrument is correlated with the explained SUA/adiposity), is usually considered to be met if the F-statistics calculated in the first stage regression is greater than 10 [36], which is true in our context. We could partly check the second assumption (i.e. the instrument is unrelated to the confounders) by examining the association between the instruments and the potential confounders (as below) that were measured, as done by others [38], [39]. We found none of the measured confounders to be significantly associated with the instruments. The third assumption (i.e. the instrument has an effect on the response of interest solely via the explained variable), is difficult to verify from the data. We compared the estimates from the OLS and 2 SLS using the Durbin-Hausman test. This process was repeated for each association of interest in the overall sample and in the sex strata. We conducted both unadjusted and adjusted analyses controlling for age, sex, smoking, alcohol use, GFR and diuretic use, covariates which can potentially influence the associations between SUA and adiposity markers. To address the possibility of confounding by population stratification, we included principal components generated from genome-wide SNPs data as covariates to the analysis. The significance level used for two-sided tests was *P*<0.05.

## Results

Table 1 summarizes the combinations that produced the best instrument for the different adiposity traits in the overall sample and by sex. Significant linear trends (either increasing or decreasing) were observed for the distribution of the phenotypes of interest across their respective genotypes or genetic scores (in the case of combined adiposity-related genetic variants) (Tables S1 and S2). Similar significant linear trends of SUA across genetic scores of adiposity-related genetic variants were noted (Table S3) but not for the distribution of adiposity markers across genotypes of *SLC2A9 rs6855911* (Table S4).

Of the 6184 participants, the range of missing genetic information varied across the different SNPs (chosen as instruments) of the *SLC2A9* and adiposity-related genes: *FTO* (range of missing data: 557–695), *MC4R* (748), *TMEM18* (650–1442) and *SLC2A9* (590–963*)*. No significant difference with regards to the phenotype of interest i.e. adiposity markers and SUA was noted between participants with and without missing genetic data. Data was also missing for the adiposity markers: weight (n = 9), body mass index (n = 9), waist circumference (n = 9) and fat mass (n = 64).

The main demographic and clinical characteristics of CoLaus participants according to sex are summarized in Table 2. Men were slightly younger than women with a mean (SD) age of 52.6 (10.8) years vs. 53.5 (10.7) years. SUA was significantly higher in men (361 (75.7) µmol/L) than in women (270.6 (67.2) µmol/L) as well as the prevalence of reported alcohol consumption and smoking. With regards to adiposity, men had significantly higher weight, BMI and waist circumference (*P*<0.001 in all) while women had higher fat mass (*P*<0.001).

**Table 2:** Demographic and clinical characteristics of CoLaus participants.

	Men (n = 2,933)		Women (n = 3,251)		
	Mean	SD	Mean	SD	*P* value
Age (years)	52.6	10.8	53.5	10.7	<0.001
Alcohol consumption^a^, %	36.1		15.7		<0.001
Current smoking^a^, %	29.3		25		<0.001
Diuretic use^a^, %	1.7		2.8		0.003
Weight (kg)	81.5	13.3	66.4	12.9	<0.001
BMI (kg/m^2^)	26.6	4.0	25.1	4.9	<0.001
WC (cm)	95.8	11.3	83.4	12.4	<0.001
Fat mass (kg)	19.8	7.6	23.4	9.5	<0.001
GFR (ml/min/1.73 m^2^)	86.7	17.4	80.7	15.2	<0.001
Serum uric acid(µmol/L)	361.1	75.7	270.6	67.2	<0.001

BMI = body mass index; GFR = estimated glomerular filtration rate (calculated according to Modification in Diet in Renal Disease equation); WC = waist circumference.

a Results are presented as percentages.

Between-group comparisons by t-test, Chi-square test or Wilcoxon ranksum test.

Table 3 displays the partial Pearson’s correlation coefficients of SUA with the selected anthropometric phenotypes, separately for men and women. SUA showed significant positive correlations with all traits (*P*<0.001). The correlations were stronger in women than in men for weight (r = 0.33 vs. r = 0.24, *P* for sex difference <0.001), BMI (r = 0.35 vs. r = 0.28, *P* = 0.002), waist circumference (r = 0.36 vs. r = 0.29, *P = *0.001), and fat mass (r = 0.35 vs. r = 0.27, *P*<0.001).

**Table 3:** Pearson’s partial correlation coefficient of adiposity markers with serum uric acid according to sex.

	Men		Women		
	r	P-value	r	P-value	P-value^a^
Weight	0.24	<0.001	0.33	<0.001	<0.001
Fat mass	0.27	<0.001	0.35	<0.001	<0.001
BMI	0.28	<0.001	0.35	<0.001	0.002
WC	0.29	<0.001	0.36	<0.001	0.001

BMI = body mass index; WC = waist circumference.

a *P* value testing the difference in correlation coefficient between men and women.

Adjusted for age, smoking, alcohol use, estimated glomerular filtration rate (GFR) and diuretic use.

We did not find any associations of the genetic variants with the other measured confounders (Table S5), thereby verifying to some extent that the instruments were independent of the measured confounders, which is an indication of the validity of the instruments.

The statistics from the first-stage regression between *SLC2A9* SNPs used as instruments and SUA presented sufficient F-statistic values (F = 170.47, 71.49 and 197.21 for *rs6855911* in overall sample, *rs7442295* in men and *rs7669607* in women respectively, Table 1). Table 4 shows the associations between SUA explained by *rs6855911* and the selected markers of adiposity (as dependent variables) in the overall sample. Both crude and adjusted analyses showed significant positive associations between SUA and all the selected adiposity markers (*P*<0.001) in the OLS regression. However, in the 2 SLS regression using instrumental variables, we observed no significant association with the adiposity traits. The results obtained from 2 SLS do not provide evidence of a causal effect of SUA on adiposity markers. This is further substantiated by the finding, in most cases, of a significant difference between the OLS and 2 SLS standardized coefficients, as shown by the P-value obtained from the Durbin-Hausman test. Similarly, conducting the same analyses but using *rs7442295* as instrument in men (Table S6) and *rs7669607* as instrument in women (Table S7) resulted in similar conclusions, with the standardized coefficients derived from 2 SLS being close to zero for all the adiposity traits.

**Table 4:** Association of SUA (using *rs6855911* from the *SLC2A9* gene as instrument) with adiposity measures (dependent variable of interest) in the overall sample.

			Ordinary least square (OLS)		2-stage least square (2 SLS)		
		N	β (95% CI)	P value_OLS_	β (95% CI)	P value_2SLS_	P value^a^
Weight	Crude	5224	0.51 (0.49, 0.53)	<0.001	0.06 (−0.08, 0.20)	0.416	<0.001
	Adjusted	5223	0.32 (0.29, 0.35)	<0.001	0.01 (−0.12, 0.14)	0.890	0.002
Fat mass	Crude	5180	0.19 (0.16, 0.21)	<0.001	0.04 (−0.11, 0.18)	0.630	0.042
	Adjusted	5179	0.37 (0.34, 0.40)	<0.001	0.05 (−0.10, 0.19)	0.521	0.004
BMI	Crude	5224	0.39 (0.37, 0.42)	<0.001	0.02 (−0.13, 0.16)	0.823	<0.001
	Adjusted	5223	0.40 (0.36, 0.43)	<0.001	−0.01 (−0.16, 0.14)	0.942	<0.001
WC	Crude	5224	0.53 (0.51, 0.56)	<0.001	0.11 (−0.03, 0.25)	0.120	<0.001
	Adjusted	5223	0.36 (0.33, 0.39)	<0.001	0.08 (−0.05, 0.21)	0.236	0.006

BMI = body mass index; SUA = serum uric acid; WC = waist circumference.

The β(95%CI) represents the association of SUA with adiposity markers as tested by the conventional epidemiological method (ordinary least square [OLS]) and by the instrumental variable analysis in a two-stage least square (2 SLS) regression (so called Mendelian randomization approach whenever the instruments are genetic variants). Similar magnitude and direction of coefficients derived from both the OLS and 2 SLS regressions suggest a causal effect of exposure (in this case SUA) on the outcome of interest (in this case adiposity). Further, a P value_2SLS_ <0.05 against the null hypothesis favors a causal effect of SUA on adiposity.

a *P* value from the Durbin-Hausman test which compares the difference between estimates derived from the OLS and 2 SLS regressions.

Results are expressed as standardized regression coefficient (β) along with 95% confidence interval (CI).

Adjusted analysis controlled for age, sex, smoking, alcohol use, estimated glomerular filtration rate (GFR) and diuretic use.

For the relationship between SUA and adiposity markers in the reverse direction, where SUA was used as the dependent variable, we obtained different combinations of SNPs that produced large enough F-statistics for the different adiposity traits separately in the overall sample, in men and in women (Table 1). Table 5 describes the coefficients derived from the OLS and 2 SLS regressions in the overall sample using combinations of adiposity-related SNPs as instrumental variables. In both crude and adjusted OLS analyses, SUA was significantly positively associated with all the selected adiposity markers (*P*<0.001) in the overall sample. The associations obtained from the 2 SLS regression were similar to the OLS regression both in magnitude (in most cases) and direction, and remained significant in the unadjusted analyses. In fat mass, the association was significant even after adjustment (*P* = 0.048). Sex-specific results are presented in Tables S8 and S9. We did not find any evidence for an interaction by sex (i.e. estimates did not significantly differ in men and in women). The direction of association with BMI in men was reversed in the 2 SLS as opposed to the OLS results although this did not result in a significant difference between the two coefficients (*P* value from Durbin-Hausman test = 0.671). Of interest is the observation that the magnitude of both the crude and adjusted coefficients was very similar in most cases, this being more apparent upon stratification by sex. The large confidence intervals in the 2 SLS associations reflect the relative weakness of the instruments. Controlling for population stratification using principal components generated from genome-wide SNPs data as covariates into the multivariable models did not produce any relevant changes in the estimates (data not shown).

**Table 5:** Association of adiposity measures (using combined SNPs from the *FTO*, *MC4R* and *TMEM18* gene as instrument) with SUA (dependent variable of interest) in the overall sample.

				Ordinary least square (OLS)		2-stage least square (2 SLS)		
	SNPs		N	β (95% CI)	*P* value_OLS_	β (95% CI)	*P* value _2 LSL_	*P* value^a^
Weight	*FTO rs1121980*+ *FTO rs1782322*+ *TMEM18 rs6755502*	Crude	5180	0.50 (0.48, 0.53)	<0.001	0.50 (0.20, 0.80)	0.001	0.947
		Adjusted	5179	0.27 (0.25, 0.30)	<0.001	0.31 −0.01, 0.62)	0.060	1.000
Fat mass	*FTO rs7193144*+ *FTO rs17823223*+ *TMEM18 rs10189761*	Crude	5396	0.19 (0.16, 0.21)	<0.001	0.49 (0.13, 0.84)	0.008	0.102
		Adjusted	5395	0.27 (0.25, 0.29)	<0.001	0.31 (0.01, 0.62)	0.048	1.000
BMI	*FTO rs1121980*+ *FTO rs2665272*+ *TMEM18 rs6755502*	Crude	5206	0.39 (0.36, 0.41)	<0.001	0.36 (0.04, 0.69)	0.026	0.900
		Adjusted	5205	0.26 (0.24, 0.29)	<0.001	0.10 (−0.22, 0.42)	0.558	0.996
WC	*FTO rs1861868*+ *FTO rs8050136*+ *TMEM18 rs6755502*	Crude	5184	0.53 (0.51, 0.55)	<0.001	0.36 (0.09, 0.64)	0.008	0.239
		Adjusted	5183	0.31 (0.28, 0.33)	<0.001	0.21 (−0.09, 0.51)	0.161	0.999

BMI = body mass index; SNP = single-nucleotide polymorphism; SUA = serum uric acid; WC = waist circumference.

The β(95%CI) represents the association of SUA with adiposity markers as tested by the conventional epidemiological method (ordinary least square [OLS]) and by the instrumental variable analysis in a two-stage least square (2 SLS) regression (so called Mendelian randomization approach whenever the instruments are genetic variants). Similar magnitude and direction of coefficients derived from both the OLS and 2 SLS regressions suggest a causal effect of exposure (in this case adiposity) on the outcome of interest (in this case SUA). Further, a P value_2SLS_ <0.05 against the null hypothesis favors a causal effect of adiposity on SUA.

a *P* value from the Durbin-Hausman test which compares the difference between estimates derived from the OLS and 2 SLS regressions.

Results are expressed as standardized regression coefficient (β) along with 95% confidence interval (CI).

Adjusted analysis controlled for age, sex, smoking, alcohol use, estimated glomerular filtration rate (GFR) and diuretic use.

## Discussion

Using a bidirectional Mendelian randomization approach in a population-based study of Caucasians aged 35 to 75 years, we tried to unravel the direction of causality between SUA and adiposity markers. SUA explained by *SLC2A9 rs6855911* in the overall sample, by *rs7442295* in men or by *rs7669607* in women, was not associated with any of the selected adiposity markers; the second-stage estimates from the instrumental variable approach were close to zero. Thus, in the present study, we found no evidence to suggest that SUA causally impacts on adiposity. By contrast, using genetic variants of the *FTO*, *MC4R* and *TMEM18* genes as instruments to explain the effect of adiposity on SUA, we observed a causal positive association of weight and fat mass with SUA in the overall sample; the association of fat mass with SUA was present in both men and women. This finding is not totally unexpected and is compatible with the hypothesis that hyperinsulinemia, a consequence of overweight and obesity, enhances renal proximal tubular reabsorption of uric acid with subsequent elevation of SUA levels [18]. Our findings are compatible with a positive causal effect of adiposity on elevated SUA. This evidence is further supported by the observation that weight reduction leads to a fall in plasma uric acid levels [25]. Considering that hyperuricemia is a strong risk factor for gout [40], [41], a potential clinical implication of our results is that weight loss should decrease, and weight gain increase, gout incidence, as recently observed in a large prospective study [42]. However, we cannot rule out the possibility that these findings could reflect a failure to fulfill the assumptions underlying Mendelian randomization.

To the best of our knowledge, this is one of the few population-based studies to use a bidirectional Mendelian randomization approach. Welsh et al. were among the first to have demonstrated the usefulness of a bidirectional Mendelian randomization approach in unraveling the directional link between adiposity and inflammation where the direction of relationship had not been otherwise proven [43]. The technique of Mendelian randomization might help to surmount the problems that are often encountered in traditional observational epidemiology. The objective of most epidemiological research is to obtain conclusions that provide causal evidence. However, this is not always possible because of the unintended noise in the data resulting from the presence of known and unknown confounders, which are often difficult to control for. In addition, there is the problem of reverse causality as it is often difficult to determine which of the two variables of interest is the cause and which is the effect. Genetic variants can be thought of exposures that have been randomly allocated at the time of gamete formation [44] and Mendelian randomization approach as a natural randomized controlled trial [45]. A bidirectional Mendelian randomization approach using genetic variants, in our context where existing evidences on the direction of causality between SUA and adiposity is conflicting and inconclusive, is a useful method.

Recent genome-wide association studies have identified the solute carrier (SLC) family 2, member 9 (*SLC2A9*) gene, encoding a putative hexose transporter, to be strongly associated with SUA [29], [34], [35], [46], including the SNP most significantly associated with SUA in this study. The *SLC2A9* gene explains a substantial proportion (about 1–6%) of variance in SUA concentration [30] and the associations between these variants and SUA have been consistently replicated across studies [29], [34], [35], [46]. Vitart et al showed that the *SLC2A9* gene has urate transport activity and found the most significant *SLC2A9* SNPs for SUA to be associated with a low fractional excretion of uric acid [29].

Conventional epidemiological studies show positive significant associations between SUA and adiposity markers (used as outcome variable although not clearly stated) like BMI [9], [47], waist-hip ratio [47] and body fat [6], [48]. Except for Masuo et al. who reported that SUA predicted subsequent weight gain [9], these studies did not clearly discuss causal associations and it is not possible to infer causality from them. The findings by Masuo et al. and by others [10]–[15] are in line with previous hypothesis of a putative causal effect of uric acid on adiposity which states that uric acid could mediate obesity and other features of the metabolic syndrome by reducing endothelial nitric oxide and decreasing insulin-mediated glucose uptake in skeletal muscle [8]. However, estimates obtained in our analysis using an instrumental variable approach did not show an association in this direction. Considering that genetic variants are not influenced by confounding and that the instruments used for these analyses were sufficiently strong, our results are certainly of interest in that they provide some evidence against a causal association in this direction.

With respect to exploring causality in the other direction, i.e. SUA could be a consequence of excess fat accumulation, we took advantage of the fact that obesity has a strong genetic component with heritability estimates ranging from 65 to 80% [49]. Unfortunately, most genetic markers identified so far only explain a very small fraction of BMI or related continuous adiposity markers, so that we had to combine multiple instruments for this analysis. The practice of combining variants from different genes into an additive genetic score to improve instruments is not uncommon [43], [50], [51] and has been shown to be an efficient linear combination of individual instruments resulting in better precision of the instrumental variable estimator. This proved practical in order to ensure sufficiently strong instruments (as evident by the F-statistic and R^2^) to fulfill the first assumption underlying the approach. However, we acknowledge that this practice can also lead to an increase in bias of the estimates [52], [53]. The current study focused on variants located within and around *FTO, MC4R* and *TMEM18* that are amongst the genes most strongly associated with obesity traits [54] and also identified in earlier meta-analyses [55]–[58], despite the fact that the variance explained by these loci is small (1–2%) [31]. Although one can argue that the instruments used for the associations in the direction of adiposity causing elevated SUA are adequate but not sufficiently strong (as illustrated by the wide confidence intervals), we observed consistent trends with weight, fat mass and waist circumference. The 2 SLS estimates did not deviate much from the OLS estimates unlike what was found when we used the *SLC2A9* variants as instruments.

The strengths of this study are its population-based design, the large sample size and accessibility to detailed and relevant information. However, our results have to be interpreted with caution since the validity of a Mendelian randomization approach in observational epidemiology relies partly on unverifiable assumptions. Some of the potential sources of residual confounding may arise due to pleiotropy and population stratification. Pleiotropy of genetic variants is difficult to address without examining all the biological pathways and this is often not possible because of the lack of understanding on the exact underlying mechanisms. However, we did not observe significant associations between any of the instruments and potential confounders suggesting that the associations are unlikely to be mediated through biological pathway involving the measured confounders. Similarly, it is reasonable to speculate that residual confounding from the association between the instruments and unmeasured confounders is minimal based on our findings of comparable crude and adjusted estimates (particularly in the direction of adiposity causing elevated SUA). We also did not find evidence of confounding by population stratification in our data.

There are also other limitations in this study. First, the adiposity-related genetic variants used as instruments were weak, resulting in the estimates having wide confidence intervals and low precision. Second, the approach used here is not the classical Mendelian Randomization approach but a slight deviation from it (which has been considered in Hernan et al [59]), since both the SUA and adiposity-related genetic variants used as instruments are not the direct gene products. Thus, there is always a risk that the proteins on the pathway work as confounders and drive the association. Third, since we included only middle-aged Caucasians, the findings may not be generalizable to other populations. Fourth, the approach of selecting the best genetic instrument in the CoLaus sample may be subject to over-fitting. Finally, an important issue is that the statistical power is, in general, not the same in both directions. In this regard, it is interesting to note that our confidence intervals of the instrumental variable analyses were in general wider when estimating a causal effect of adiposity on SUA than when estimating a causal effect of SUA on adiposity (recall that since all variables are standardized, the effects are expressed on a similar scale, which allows such a comparison). This means that we had more power in the direction where we could not find a significant causal effect than in the direction where we found some significant causal effects (this being consistent with the fact that we had a stronger instrument in the former direction). Thus, our non-significant causal effects of SUA on adiposity may not only be due to a lack of power.

In conclusion, using a bidirectional Mendelian randomization approach, our findings suggest that elevated SUA is a consequence rather than a cause of elevated adiposity. To our knowledge, this is the first study in which the relationship between SUA and adiposity has been explored using genetic tools. While future studies are essential to confirm these findings, our observations may shed some light on the uncertainty underlying this pathophysiological link and highlight the usefulness of the bidirectional Mendelian randomization approach to decipher the direction of causality.

## Supporting Information

Table S1 **Genotype distribution of adiposity markers and SUA across the adiposity-related and SUA-related SNPs respectively.**
(DOC)

Table S2 **Distribution of adiposity markers across scores of adiposity-related SNPs.**
(DOC)

Table S3 **Distribution of SUA across scores of adiposity-related SNPs.**
(DOC)

Table S4 **Distribution of adiposity markers across genotypes of** ***SLC2A9 rs6855911.***
(DOC)

Table S5 **Association between the SNP/SNP scores with potential confounders.**
(DOC)

Table S6 **Association of SUA (using** ***rs7442295*** **from the** ***SLC2A9*** **gene as instrument) with adiposity measures (dependent variable of interest) in men.**
(DOC)

Table S7 **Association of SUA (using** ***rs7669607*** **from the** ***SLC2A9*** **gene as instrument) with adiposity measures (dependent variable of interest) in women.**
(DOC)

Table S8 **Association of adiposity measures (using combined SNPs from the** ***FTO*** **,** ***MC4R*** **and** ***TMEM18*** **gene as instrument) with SUA (dependent variable of interest) in men.**
(DOC)

Table S9 **Association of adiposity measures (using combined SNPs from the** ***FTO*** **,** ***MC4R*** **and** ***TMEM18*** **gene as instrument) with SUA (dependent variable of interest) in women.**
(DOC)
