# Supplementary material for: Serum Uric Acid and Adiposity: Deciphering Causality Using a Bidirectional Mendelian Randomization Approach
Source: PLoS One. 2012 Jun 19;7(6):e39321. doi: 10.1371/journal.pone.0039321 (PMC3378571; doi:10.1371/journal.pone.0039321)
Supplement: Table S1 — Genotype distribution of adiposity markers and SUA across the adiposity-related and SUA-related SNPs respectively. (DOC) [file pone.0039321.s001.doc]

**Table S1: Genotype distribution of adiposity markers and SUA across the adiposity-related and SUA-related SNPs respectively**

| **Phenotype** | **SNPs** |  | **mean (SD)** |  | **P-trend** |
| --- | --- | --- | --- | --- | --- |
| Weight (kg) | *FTO rs1121980* | CC (n=1778) | CT (n=2759) | TT (n=1091) |  |
|  |  | 72.5 (14.9) | 73.6 (15.2) | 75.0 (15.3) | <0.001 |
|  | *FTO rs17823223* | CC (n=4019) | CT (n=1437) | TT (n=141) |  |
|  |  | 73.7 (15.2) | 73.0 (14.7) | 71.3 (14.2) | 0.027 |
|  | *TMEM18 rs6755502* | AA (n=154) | AG (n=1602) | GG (n=3457) |  |
|  |  | 72.2 (15.1) | 72.7 (15.0) | 74.0 (15.1) | 0.002 |
| Fat mass (kg) | *FTO rs7193144* | AA (n=1947) | AG (n=2696) | GG (n=881) |  |
|  |  | 21.1 (8.7) | 22.0 (8.9) | 22.4 (8.9) | <0.001 |
|  | *FTO rs17823223* | CC (n=3985) | CT (n=1425) | TT (n=141) |  |
|  |  | 21.9 (8.9) | 21.3 (8.4) | 20.3 (8.0) | 0.042 |
|  | *TMEM18 rs10189761* | AA (n=3642) | AT (n=1666) | TT (n=181) |  |
|  |  | 21.9 (8.9) | 21.4 (8.7) | 21.1 (9.1) | 0.011 |
| BMI (kg/m^2^) | *FTO rs1121980* | CC (n=1778) | CT (n=2759) | TT (n=1091) |  |
|  |  | 25.5 (4.5) | 25.8 (4.5) | 26.2 (4.6) | <0.001 |
|  | *FTO rs26665272* | CC (n=1363) | CT (n=2832) | TT (n=1431) |  |
|  |  | 26.1 (4.6) | 25.8 (4.5) | 25.6 (4.6) | 0.021 |
|  | *TMEM18 rs6755502* | AA (n=154) | AG (n=1602) | GG (n=3457) |  |
|  |  | 25.3 (4.4) | 25.6 (4.6) | 25.9 (4.6) | <0.001 |
| WC (cm) | *FTO rs1861868* | AA (n=1491) | AG (n=2825) | GG (n=1298) |  |
|  |  | 89.9 (13.3) | 89.2 (13.3) | 88.5 (13.6) | 0.004 |
|  | *FTO rs8050136* | AA (n=924) | AC (n=2724) | CC (n=1969) |  |
|  |  | 90.7 (13.4) | 89.6 (13.5) | 88.1 (13.2) | <0.001 |
|  | *TMEM18 rs6755502* | AA (n=154) | AG (n=1602) | GG (n=3457) |  |
|  |  | 87.9 (13.3) | 88.6 (13.6) | 89.6 (13.3) | 0.002 |
| SUA (µmol/L) | *SLC2A9 rs6855911* | AA (n=2684) | AG (n=2142) | GG (n=398) |  |
|  |  | 325.5 (82.3) | 303.5 (83.6) | 275.4 (83.4) | <0.001 |

Results expressed as numbers and mean (standard deviation).

BMI= body mass index; WC= waist circumference; SNP=single-nucleotide polymorphism; SUA=serum uric acid.
