# Supplementary material for: Serum Uric Acid and Adiposity: Deciphering Causality Using a Bidirectional Mendelian Randomization Approach
Source: PLoS One. 2012 Jun 19;7(6):e39321. doi: 10.1371/journal.pone.0039321 (PMC3378571; doi:10.1371/journal.pone.0039321)
Supplement: Table S2 — Distribution of adiposity markers across scores of adiposity-related SNPs. (DOC) [file pone.0039321.s002.doc]

**Table S2: Distribution of adiposity markers across scores of adiposity-related SNPs**

| **Phenotype** | **SNP combination** | **0-2*** | **3** | **4** | **5** | **6** | **P-trend** |
| --- | --- | --- | --- | --- | --- | --- | --- |
| Weight (kg) | *FTO rs1121980* + *FTO rs17823223* + *TMEM18 rs6755502* | 259 | 946 | 1912 | 1600 | 464 |  |
|  |  | 71.7(15.1) | 71.4(14.1) | 73.7(15.3) | 74.1(15.1) | 75.8(15.5) | <0.001 |
| Fat mass (kg) | *FTO rs7193144* + *FTO rs17823223* + *TMEM18 rs10189761* | 298 | 1047 | 2067 | 1573 | 412 |  |
|  |  | 20.0(7.8) | 21.2(8.5) | 21.5(8.9) | 22.3(8.8) | 22.7(9.3) | <0.001 |
| BMI (kg/m^2^) | *FTO rs1121980* + *FTO rs2665272* + *TMEM18 rs6755502* | 966 | 1617 | 1630 | 851 | 143 |  |
|  |  | 25.3(4.6) | 25.7(4.6) | 25.9(4.4) | 26.3(4.8) | 26.2(4.5) | <0.001 |
| WC (cm) | *FTO rs1861868* + *FTO rs8050136* + *TMEM18 rs6755502* | 1161 | 1429 | 1527 | 808 | 260 |  |
|  |  | 87.4(13.2) | 89.1(13.6) | 89.8(13.2) | 90.1(13.4) | 91.9(13.4) | <0.001 |

Results are expressed as numbers and mean (standard deviation).

BMI= body mass index; WC=waist circumference; SNP=single-nucleotide polymorphism.

*Participants having scores 0, 1 and 2 were combined into one category since the numbers within these individual scores were small.
