# Supplementary material for: Serum Uric Acid and Adiposity: Deciphering Causality Using a Bidirectional Mendelian Randomization Approach
Source: PLoS One. 2012 Jun 19;7(6):e39321. doi: 10.1371/journal.pone.0039321 (PMC3378571; doi:10.1371/journal.pone.0039321)
Supplement: Table S3 — Distribution of SUA across scores of adiposity-related SNPs. (DOC) [file pone.0039321.s003.doc]

**Table S3: Distribution of SUA across scores of adiposity-related SNPs**

|  | **SNP combination** | **Instruments for** | **0-2*** | **3** | **4** | **5** | **6** | **P-trend** |
| --- | --- | --- | --- | --- | --- | --- | --- | --- |
| SUA | *FTO rs1121980* + *FTO rs17823223* + *TMEM18 rs6755502* | Weight | 259 | 946 | 1912 | 1600 | 464 |  |
|  |  |  | 312(79.9) | 306(84.2) | 315(84.6) | 315(84.9) | 321(87.2) | 0.018 |
| SUA | *FTO rs7193144* + *FTO rs17823223* + *TMEM18 rs10189761* | Fat mass | 298 | 1047 | 2067 | 1573 | 412 |  |
|  |  |  | 310(77.7) | 307(84.4) | 313(84.2) | 316(85.0) | 320(86.8) | 0.020 |
| SUA | *FTO rs1121980* + *FTO rs2665272* + *TMEM18 rs6755502* | BMI | 966 | 1617 | 1630 | 851 | 143 |  |
|  |  |  | 308(84.2) | 314(83.7) | 314(83.8) | 318(88.7) | 309(82.5) | 0.034 |
| SUA | *FTO rs1861868* + *FTO rs8050136* + *TMEM18 rs6755502* | WC | 1161 | 1429 | 1527 | 808 | 260 |  |
|  |  |  | 311(82.1) | 309(85.0) | 318(85.2) | 314(84.5) | 322(89.7) | 0.077 |

Results are expressed as numbers and mean (standard deviation).

SUA= serum uric acid; BMI= body mass index; WC=waist circumference; SNP=single-nucleotide polymorphism.

*Participants having scores 0, 1 and 2 were combined into one category since the numbers within these individual scores were small.
