# Supplementary material for: Serum Uric Acid and Adiposity: Deciphering Causality Using a Bidirectional Mendelian Randomization Approach
Source: PLoS One. 2012 Jun 19;7(6):e39321. doi: 10.1371/journal.pone.0039321 (PMC3378571; doi:10.1371/journal.pone.0039321)
Supplement: Table S4 — Distribution of adiposity markers across genotypes of SLC2A9 rs6855911. (DOC) [file pone.0039321.s004.doc]

**Table S4: Distribution of adiposity markers across genotypes of *SLC2A9 rs6855911***

|  | ***SLC2A9 rs6855911*** | | |  |
| --- | --- | --- | --- | --- |
| **Phenotype** | **AA (n=2684)** | **AG (n=2142)** | **GG (n=398)** | **P-trend** |
| SUA (µmol/L) | 325 (82.3) | 303 (83.6) | 275 (83.4) | <0.001 |
| Weight (kg) | 73.7 (15.2) | 73.0 (15.0) | 73.9 (15.1) | 0.356 |
| Fat mass (kg) | 21.7 (8.9) | 21.6 (8.6) | 21.6 (9.4) | 0.699 |
| BMI (kg/m^2^) | 25.8 (4.6) | 25.8 (4.5) | 25.8 (4.7) | 0.861 |
| WC (cm) | 89.5 (13.5) | 88.8 (13.2) | 89.2 (13.8) | 0.172 |

Results are expressed as mean (standard deviation).

SUA= serum uric acid; BMI= body mass index; WC=waist circumference.
