# Supplementary material for: Serum Uric Acid and Adiposity: Deciphering Causality Using a Bidirectional Mendelian Randomization Approach
Source: PLoS One. 2012 Jun 19;7(6):e39321. doi: 10.1371/journal.pone.0039321 (PMC3378571; doi:10.1371/journal.pone.0039321)
Supplement: Table S5 — Association between the SNP/SNP scores with potential confounders. (DOC) [file pone.0039321.s005.doc]

**Table S5: Association between the SNP/SNP scores with potential confounders**

| **Potential confounders** | **β coefficients** | **P value** |
| --- | --- | --- |
| *Age* |  |  |
| *FTO rs1121980* + *FTO rs17823223* + *TMEM18 rs6755502* | -0.09 | 0.550 |
| *FTO rs7193144* + *FTO rs17823223* + *TMEM18 rs10189761* | -0.02 | 0.900 |
| *FTO rs1121980* + *FTO rs2665272* + *TMEM18 rs6755502* | 0.10 | 0.469 |
| *FTO rs1861868* + *FTO rs8050136* + *TMEM18 rs6755502* | 0.00 | 0.997 |
| *SLC2A9 rs6855911* | -0.04 | 0.858 |
| *Smoking* |  |  |
| *FTO rs1121980* + *FTO rs17823223* + *TMEM18 rs6755502* | 0.00 | 0.676 |
| *FTO rs7193144* + *FTO rs17823223* + *TMEM18 rs10189761* | 0.00 | 0.831 |
| *FTO rs1121980* + *FTO rs2665272* + *TMEM18 rs6755502* | 0.00 | 0.898 |
| *FTO rs1861868* + *FTO rs8050136* + *TMEM18 rs6755502* | -0.01 | 0.396 |
| *SLC2A9 rs6855911* | 0.01 | 0.346 |
| *Alcohol* |  |  |
| *FTO rs1121980* + *FTO rs17823223* + *TMEM18 rs6755502* | 0.00 | 0.598 |
| *FTO rs7193144* + *FTO rs17823223* + *TMEM18 rs10189761* | 0.00 | 0.659 |
| *FTO rs1121980* + *FTO rs2665272* + *TMEM18 rs6755502* | -0.01 | 0.268 |
| *FTO rs1861868* + *FTO rs8050136* + *TMEM18 rs6755502* | 0.00 | 0.573 |
| *SLC2A9 rs6855911* | 0.01 | 0.592 |
| *GFR* |  |  |
| *FTO rs1121980* + *FTO rs17823223* + *TMEM18 rs6755502* | 0.23 | 0.294 |
| *FTO rs7193144* + *FTO rs17823223* + *TMEM18 rs10189761* | 0.10 | 0.643 |
| *FTO rs1121980* + *FTO rs2665272* + *TMEM18 rs6755502* | 0.00 | 0.984 |
| *FTO rs1861868* + *FTO rs8050136* + *TMEM18 rs6755502* | -0.11 | 0.628 |
| *SLC2A9 rs6855911* | 0.58 | 0.110 |
| *Diuretic use* |  |  |
| *FTO rs1121980* + *FTO rs17823223* + *TMEM18 rs6755502* | 0.00 | 0.813 |
| *FTO rs7193144* + *FTO rs17823223* + *TMEM18 rs10189761* | 0.00 | 0.863 |
| *FTO rs1121980* + *FTO rs2665272* + *TMEM18 rs6755502* | 0.00 | 0.342 |
| *FTO rs1861868* + *FTO rs8050136* + *TMEM18 rs6755502* | 0.00 | 0.851 |
| *SLC2A9 rs6855911* | 0.00 | 0.919 |
| *C-reactive protein* |  |  |
| *FTO rs1121980* + *FTO rs17823223* + *TMEM18 rs6755502* | -0.02 | 0.642 |
| *FTO rs7193144* + *FTO rs17823223* + *TMEM18 rs10189761* | -0.03 | 0.524 |
| *FTO rs1121980* + *FTO rs2665272* + *TMEM18 rs6755502* | 0.01 | 0.773 |
| *FTO rs1861868* + *FTO rs8050136* + *TMEM18 rs6755502* | -0.06 | 0.195 |
| *SLC2A9 rs6855911* | 0.07 | 0.356 |

GFR= glomerular filtration rate estimated using the Modification of the Diet in Renal Disease (MDRD) formula.
