# Supplementary material for: Serum Uric Acid and Adiposity: Deciphering Causality Using a Bidirectional Mendelian Randomization Approach
Source: PLoS One. 2012 Jun 19;7(6):e39321. doi: 10.1371/journal.pone.0039321 (PMC3378571; doi:10.1371/journal.pone.0039321)
Supplement: Table S6 — Association of SUA (using rs7442295 from the SLC2A9 gene as instrument) with adiposity measures (dependent variable of interest) in men. (DOC) [file pone.0039321.s006.doc]

**Table S6: Association of SUA (using *rs7442295* from the *SLC2A9*** gene as instrument) with adiposity measures (dependent variable of interest) in men

|  |  |  | **Ordinary least square(OLS)** | | **2-stage least square (2SLS)** | |  |
| --- | --- | --- | --- | --- | --- | --- | --- |
|  |  | **N** | **β (95% CI)** | ***P* value_OLS_** | **β (95% CI)** | ***P* value_2SLS_** | ***P* value^a^** |
| Weight | Crude | 2629 | 0.26(0.23,0.30) | <0.001 | 0.05(-0.18,0.28) | 0.666 | 0.062 |
|  | Adjusted | 2628 | 0.25(0.22,0.29) | <0.001 | 0.01(-0.23,0.25) | 0.956 | 0.653 |
| Fat mass | Crude | 2608 | 0.30(0.26,0.34) | <0.001 | 0.11(-0.12,0.34) | 0.341 | 0.094 |
|  | Adjusted | 2607 | 0.27(0.23,0.30) | <0.001 | 0.02(-0.20,0.25) | 0.838 | 0.617 |
| BMI | Crude | 2629 | 0.30(0.26,0.33) | <0.001 | 0.08(-0.14,0.31) | 0.457 | 0.056 |
|  | Adjusted | 2628 | 0.29(0.25,0.33) | <0.001 | 0.04(-0.20,0.27) | 0.762 | 0.611 |
| WC | Crude | 2628 | 0.31(0.28,0.35) | <0.001 | 0.08(-0.14,0.31) | 0.464 | 0.039 |
|  | Adjusted | 2627 | 0.29(0.26,0.33) | <0.001 | 0.02(-0.22,0.25) | 0.898 | 0.463 |

BMI=body mass index; SUA=serum uric acid; WC=waist circumference.

The β(95%CI) represents the association of SUA with adiposity markers as tested by the conventional epidemiological method (ordinary least square [OLS]) and by the instrumental variable analysis in a two-stage least square (2SLS) regression (so called Mendelian randomization approach whenever the instruments are genetic variants). Similar magnitude and direction of coefficients derived from both the OLS and 2SLS regressions suggest a causal effect of exposure (in this case SUA) on the outcome of interest (in this case adiposity). Further, a P value_2SLS_ < 0.05 against the null hypothesis favors a causal effect of SUA on adiposity.

^a^ *P* value from the Durbin-Hausman test which compares the difference between estimates derived from the OLS and 2SLS regressions.

Results are expressed as standardized regression coefficient (β) along with 95% confidence interval (CI).

Adjusted analysis controlled for age, smoking, alcohol use, estimated glomerular filtration rate (GFR) and diuretic use.
