# Supplementary material for: Serum Uric Acid and Adiposity: Deciphering Causality Using a Bidirectional Mendelian Randomization Approach
Source: PLoS One. 2012 Jun 19;7(6):e39321. doi: 10.1371/journal.pone.0039321 (PMC3378571; doi:10.1371/journal.pone.0039321)
Supplement: Table S8 — Association of adiposity measures (using combined SNPs from the FTO , MC4R and TMEM18 gene as instrument) with SUA (dependent variable of interest) in men. (DOC) [file pone.0039321.s008.doc]

**Table S8: Association of adiposity measures (using combined SNPs from the *FTO*, *MC4R* and *TMEM18*** gene as instrument) with SUA (dependent variable of interest) in men

|  |  |  |  | **Ordinary least square (OLS)** | | **2-stage least square (2SLS)** | |  |
| --- | --- | --- | --- | --- | --- | --- | --- | --- |
|  | **SNPs** |  | **N** | **β (95% CI)** | ***P* value_OLS_** | **β (95% CI)** | ***P* value_2SLS_** | ***P* value^a^** |
| Weight | *FTO rs7193144* + *FTO rs17823223* + *FTO rs2192872* | Crude | 2592 | 0.27(0.23,0.30) | <0.001 | 0.36(-0.12,0.85) | 0.140 | 0.690 |
|  |  | Adjusted | 2591 | 0.24(0.21,0.28) | <0.001 | 0.28(-0.22,0.78) | 0.268 | 1.000 |
| Fat mass | *FTO rs7193144* + *FTO rs16945088* + *FTO rs17823223* | Crude | 2549 | 0.30(0.27,0.34) | <0.001 | 0.49(0.10,0.89) | 0.014 | 0.345 |
|  |  | Adjusted | 2548 | 0.29(0.25,0.33) | <0.001 | 0.44(-0.01,0.88) | 0.055 | 0.999 |
| BMI | *FTO rs7193144* + *FTO rs6499658* + *TMEM18 rs2683992* | Crude | 2421 | 0.31(0.27,0.35) | <0.001 | -0.14(-0.58,0.30) | 0.532 | 0.045 |
|  |  | Adjusted | 2420 | 0.28(0.24,0.32) | <0.001 | -0.16(-0.59,0.27) | 0.462 | 0.671 |
| WC | *FTO rs8050136* + *FTO rs8053740* + *MC4R rs17066829* | Crude | 2566 | 0.31(0.28,0.35) | <0.001 | 0.34(-0.11,0.80) | 0.141 | 0.898 |
|  |  | Adjusted | 2565 | 0.29(0.25,0.33) | <0.001 | 0.34((-0.13,0.80) | 0.154 | 1.000 |

BMI=body mass index; SNP=single-nucleotide polymorphism; SUA=serum uric acid; WC=waist circumference.

The β(95%CI) represents the association of SUA with adiposity markers as tested by the conventional epidemiological method (ordinary least square [OLS]) and by the instrumental variable analysis in a two-stage least square (2SLS) regression (so called Mendelian randomization approach whenever the instruments are genetic variants). Similar magnitude and direction of coefficients derived from both the OLS and 2SLS regressions suggest a causal effect of exposure (in this case adiposity) on the outcome of interest (in this case SUA). Further, a P value_2SLS_ < 0.05 against the null hypothesis favors a causal effect of SUA on adiposity.

^a^ *P* value from the Durbin-Hausman test which compares the difference between estimates derived from the OLS and 2SLS regressions.

Results are expressed as standardized regression coefficient (β) along with 95% confidence interval (CI).

Adjusted analysis controlled for age, sex, smoking, alcohol use, estimated glomerular filtration rate (GFR) and diuretic use.
