# Supplementary material for: Serum Uric Acid and Adiposity: Deciphering Causality Using a Bidirectional Mendelian Randomization Approach
Source: PLoS One. 2012 Jun 19;7(6):e39321. doi: 10.1371/journal.pone.0039321 (PMC3378571; doi:10.1371/journal.pone.0039321)
Supplement: Table S9 — Association of adiposity measures (using combined SNPs from the FTO , MC4R and TMEM18 gene as instrument) with SUA (dependent variable of interest) in women. (DOC) [file pone.0039321.s009.doc]

**Table S9: Association of adiposity measures (using combined SNPs from the *FTO*, *MC4R* and *TMEM18*** gene as instrument) with SUA (dependent variable of interest) in women

|  |  |  |  | **Ordinary least square (OLS)** | | **2-stage least square (2SLS)** | |  |
| --- | --- | --- | --- | --- | --- | --- | --- | --- |
|  | **SNPs** |  | **N** | **β (95% CI)** | ***P* value_OLS_** | **β (95% CI)** | ***P* value_2SLS_** | ***P* value^a^** |
| Weight | *FTO rs9939973* + *FTO rs836994* + *TMEM18 rs6755502* | Crude | 2712 | 0.35(0.32,0.39) | <0.001 | 0.19(-0.21,0.60) | 0.344 | 0.441 |
|  |  | Adjusted | 2712 | 0.31(0.27,0.34) | <0.001 | 0.22(-0.15,0.58) | 0.241 | 0.999 |
| Fat mass | *FTO rs1121980* + *FTO rs17823223* + *TMEM18 rs7585056* | Crude | 2812 | 0.42(0.38,0.45) | <0.001 | 0.41(0.02,0.80) | 0.038 | 0.988 |
|  |  | Adjusted | 2812 | 0.34(0.31,0.38) | <0.001 | 0.39(-0.01,0.80) | 0.055 | 1.000 |
| BMI | *FTO rs2540769* + *FTO rs2665272* + *TMEM18 rs2860323* | Crude | 2555 | 0.37(0.34,0.41) | <0.001 | 0.33(-0.07,0.72) | 0.104 | 0.819 |
|  |  | Adjusted | 2555 | 0.33(0.29,0.36) | <0.001 | 0.08(-0.36,0.50) | 0.731 | 0.971 |
| WC | *FTO rs1121980* + *FTO rs2665272* + *TMEM18 rs7571872* | Crude | 2489 | 0.41(0.37,0.44) | <0.001 | 0.22(-0.09,0.55) | 0.176 | 0.256 |
|  |  | Adjusted | 2489 | 0.34(0.31,0.37) | <0.001 | 0.12(-0.24,0.47) | 0.520 | 0.953 |

BMI=body mass index; SNP=single-nucleotide polymorphism; SUA=serum uric acid; WC=waist circumference.

The β(95%CI) represents the association of SUA with adiposity markers as tested by the conventional epidemiological method (ordinary least square [OLS]) and by the instrumental variable analysis in a two-stage least square (2SLS) regression (so called Mendelian randomization approach whenever the instruments are genetic variants). Similar magnitude and direction of coefficients derived from both the OLS and 2SLS regressions suggest a causal effect of exposure (in this case adiposity) on the outcome of interest (in this case SUA). Further, a P value_2SLS_ < 0.05 against the null hypothesis favors a causal effect of SUA on adiposity.

^a^ *P* value from the Durbin-Hausman test which compares the difference between estimates derived from the OLS and 2SLS regressions.

Results are expressed as standardized regression coefficient (β) along with 95% confidence interval (CI).

Adjusted analysis controlled for age, sex, smoking, alcohol use, estimated glomerular filtration rate (GFR) and diuretic use.
